# Cold atmospheric plasma as a potential tool for multiple myeloma treatment

**DOI:** 10.18632/oncotarget.24649

**Published:** 2018-04-06

**Authors:** Dehui Xu, Yujing Xu, Qingjie Cui, Dingxin Liu, Zhijie Liu, Xiaohua Wang, Yanjie Yang, Miaojuan Feng, Rong Liang, Hailan Chen, Kai Ye, Michael G. Kong

**Affiliations:** ^1^ State Key Laboratory of Electrical Insulation and Power Equipment, Center for Plasma Biomedicine, Xi’an Jiaotong University, Xi’an, Shaanxi, 710049, P.R. China; ^2^ The School of Life Science and Technology, Xi’an Jiaotong University, Xi’an, Shaanxi, 710049, P.R. China; ^3^ Department of Cardiovascular Medicine, First Affiliated Hospital of the Medical School, Xi’an Jiaotong University, Xi’an, Shaanxi, 710061, P.R. China; ^4^ Department of Hematology, Xijing Hospital, Fourth Military Medical University, Xi’an, Shaanxi, 710032, P.R. China; ^5^ Frank Reidy Center for Bioelectrics, Old Dominion University, Norfolk, VA, 23508, USA; ^6^ School of Electronic and Information Engineering, Xi’an Jiaotong University, Xi’an, Shaanxi, 710049, P.R. China; ^7^ First Affiliated Hospital of the Medical School, Xi’an Jiaotong University, Xi’an, Shaanxi, 710061, P.R. China; ^8^ Department of Electrical and Computer Engineering, Old Dominion University, Norfolk, VA, 23529, USA

**Keywords:** multiple myeloma, cold atmospheric plasma, reactive oxygen species, CD95, selective inactivation

## Abstract

Multiple myeloma (MM) is a fatal and incurable hematological malignancy thus new therapy need to be developed. Cold atmospheric plasma, a new technology that could generate various active species, could efficiently induce various tumor cells apoptosis. More details about the interaction of plasma and tumor cells need to be addressed before the application of gas plasma in clinical cancer treatment. In this study, we demonstrate that He+O_2_ plasma could efficiently induce myeloma cell apoptosis through the activation of CD95 and downstream caspase cascades. Extracellular and intracellular reactive oxygen species (ROS) accumulation is essential for CD95-mediated cell apoptosis in response to plasma treatment. Furthermore, p53 is shown to be a key transcription factor in activating CD95 and caspase cascades. More importantly, we demonstrate that CD95 expression is higher in tumor cells than in normal cells in both MM cell lines and MM clinical samples, which suggests that CD95 could be a favorable target for plasma treatment as it could selectively inactivate myeloma tumor cells. Our results illustrate the molecular details of plasma induced myeloma cell apoptosis and it shows that gas plasma could be a potential tool for myeloma therapy in the future.

## INTRODUCTION

Multiple myeloma (MM), the second most common hematological malignancy characterized by the accumulation of malignant plasma cells (PCs) in the bone marrow (BM) of patients, results in the production of monoclonal immunoglobulin (also known as ‘M-protein’) and substantial immune-suppression and end-organ damage such as anemia, thrombocytopenia, renal failure, and bone disease [1]. In nonrandomized MM clinical trials, high-dose chemotherapy is recommended with an autologous transplant, which results in the highest response rates [2, 3]. However, despite great progress in novel anti-myeloma drugs and therapies, most of the patients relapse and become resistant to chemotherapy [4], which makes MM a fatal and incurable hematological malignancy. Regulation of redox homeostasis is one strategy for cancer treatment, as long-term ROS simulation plays an important role in cancer development through oxidative DNA damage and gene mutations [5, 6]. Radiation and several chemotherapy drugs can produce extracellular and intracellular ROS, thus disrupting redox homeostasis, which can induce tumor cell apoptosis [7, 8]. Cell apoptosis are mainly conducted by three pathways: mitochondrial pathway, extrinsic pathway such as death receptors (DR) and tumor necrosis factor receptor (TNF), endoplasmic reticulum (ER) stress pathway [9, 10]. In this study, we investigated whether CD95, a key death receptor that is pivotal for the induction of tumor cell apoptosis and is a major target in cancer therapy [11, 12], is involved in plasma induced cell apoptosis. It was reported that CD95 is widely expressed in patient myeloma cells and myeloma cell lines [13, 14]. Anti-CD95 antibodies could directly target CD95; however, only some samples exhibited cell apoptosis [15]. ROS accumulation could also regulate CD95, thus activating CD95-mediated tumor cell apoptosis [16, 17]. In addition to radio- and chemotherapies, a new technology called cold atmospheric plasma (CAP) could provide controllable exogenous reactive oxygen and nitrogen species [18, 19]. Plasmas are ionized gases, comprising a complex mixture of charged particles, neutral gas molecules, UV radiation, electric fields and reactive species [20]. Owing to technological advancements, it is now possible to generate plasmas at atmospheric pressure and room temperature (e.g., CAP), which enables the application of plasma in a range of biomedical processes such as wound healing, disinfection and cancer treatment [21, 22]. Most plasma devices could be divided into two types: plasma jet and Dielectric Barrier Discharger (DBD) [23–25]. CAP is a favorable source of reactive oxygen and nitrogen species, and it has been found to be efficient for killing tumor cells in various types of cancer, including lung cancer, leukemia, intestinal cancer, melanoma, cervical cancer, glioma, pancreatic cancer et al. [26–29]. The induction of apoptosis in cancer cells has been widely reported, and the mechanism of plasma-induced apoptosis is increasingly being understood [30–32]. It is reported that plasma could affect various cancer cell signaling such as AGP-ROS, MAPK, p53 and PI3K/AKT pathway and induced cell apoptosis [33]. In this study, we first demonstrated that CD95 was involved in plasma-induced cancer cell apoptosis. Up-regulation of CD95 was positively correlated with the accumulation of extracellular and intracellular ROS. By chromatin immune-precipitation (ChIP) assay, we demonstrated that p53, which was up-regulated by plasma treatment, could bind to the CD95 promoter region and increase CD95 expression, resulting in the activation of caspase signaling and induction of apoptosis. Furthermore, because of the differential expression of CD95 in normal and myeloma cells, plasma could selectively inactivate tumor cells in myeloma cell lines and patient samples. Our results provide a new strategy for potential myeloma therapy by targeting CD95 with cold atmospheric plasma.

## RESULTS

### Characteristics of plasma generation

**Figure 1:**
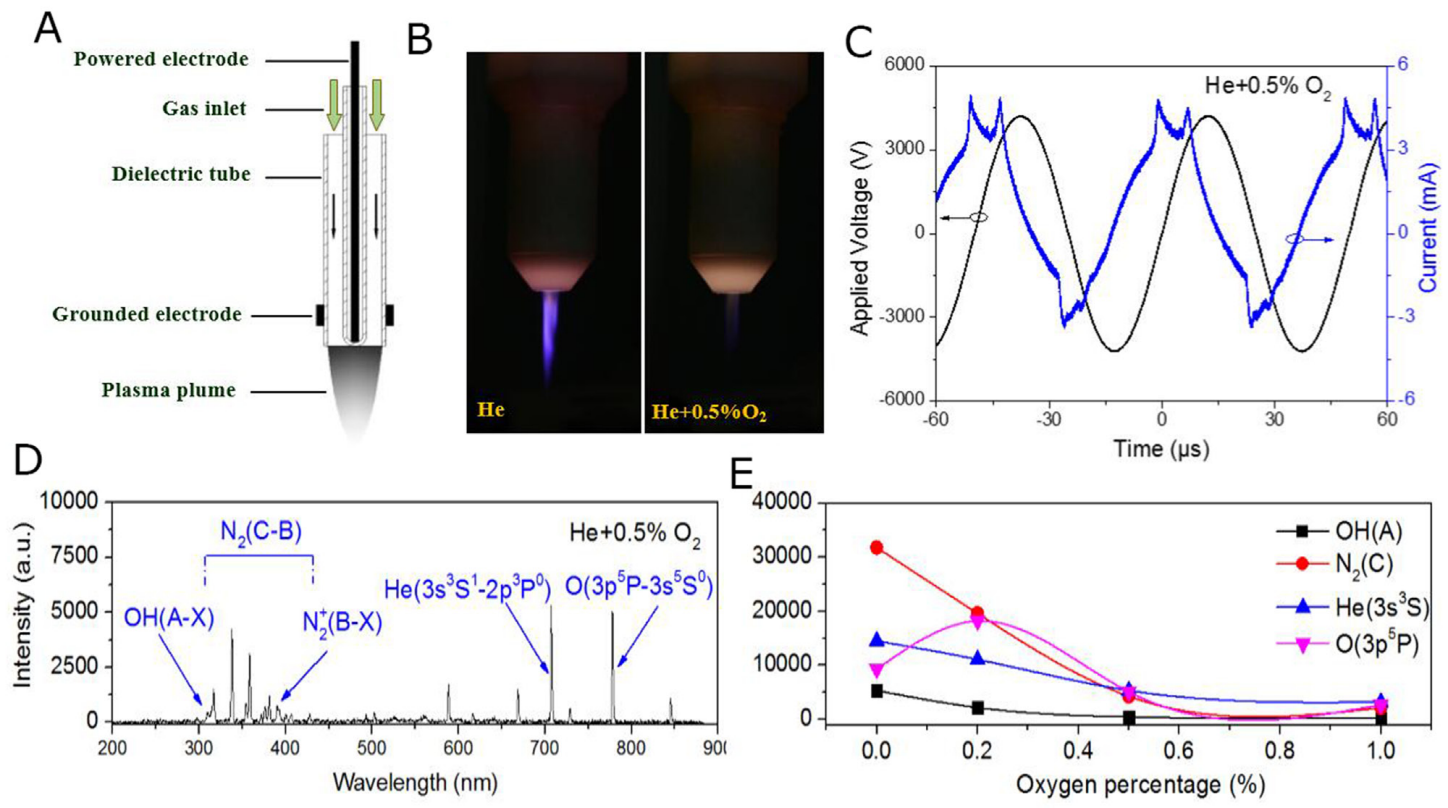
Characteristics of the plasma generation system **(A)** Schematic representation of the plasma jet. **(B)** Photographs of He plasma and He+O_2_ plasmas. **(C)** Monitoring of the applied voltage and current during He+O_2_ plasma generation. **(D)** Emission spectra of different He+O_2_ plasmas detected by the spectrometer. Some unique spectral lines (OH, N_2_, He and O) in the He+0.5% O_2_ plasmas are marked. **(E)** The intensity of several characteristic spectral lines are shown with varying O_2_ percentage in the working gas.

He plasma was generated at the voltage of 10 kHz/8 kV with a He gas flow of 2 SLM. Additional O_2_ was mixed in to produce more ROS. (Figure 1A and 1B) show the structure of the plasma jet and photographs of He/He+O_2_ plasmas. Additional O_2_ decreased the intensity of the plasma and changed the plasma plume from purple to gray. (Figure 1C) shows the corresponding applied voltage and current during He+O_2_ plasma generation. To investigate the different reactive species in the plasma, we used a spectrometer to measure the emission spectra. As shown in (Figure 1D), there were several spectral lines (e.g., OH (A) 309 nm, N_2_ (C) 337 nm, N_2_^+^ (B) 391 nm, He (3s^3^S^1^) 706 nm and O (3p^5^P) 777 nm) present in the plasma and they were marked according to the literature [34, 35]. The intensity of the plasma components gradually decreased when the percentage of mixed O_2_ was increased from 0% to 1% (Figure 1E).

### Decrease in cell viability and induction of apoptosis after plasma treatment for different durations

**Figure 2:**
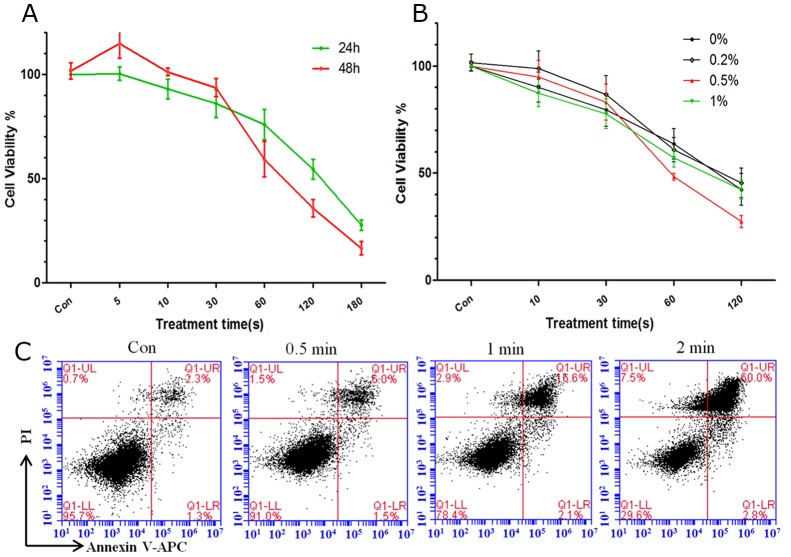
Cell viability and apoptosis after plasma treatment for different durations **(A)** Analysis of cell viability 24 h and 48 h after He plasma treatment for different durations. **(B)** Analysis of cell viability 24 h after He+O_2_ plasma for different durations. **(C)** Cell apoptosis analyzed by flow cytometry after He+0.5%O_2_ plasma treatment for 0.5, 1, and 2 min.

We treated LP-1 MM cells with CAP and investigated the biological effects of plasma. CellTiter-Glo assay showed that the cell viability 24 h and 48 h after He plasma treatment gradually decreased in a time-dependent manner (Figure 2A). Next, we added O_2_ to He to generate He+O_2_ plasma, which could produce more ROS. We added O_2_ at 0.2%, 0.5% and 1% of the total mixture. Because O_2_ is a electronegative gas, a mixture with greater than 1% of O_2_ will eliminate plasma generation. (Figure 2B) shows the cell viability 24 h after the application of the various compositions of He+O_2_ plasma. The addition of 0.2% O_2_ didn't promote LP-1 cell death compared to He+0% O_2_, and the cell viability of He+0.5% O_2_ treatment was sharply decreased and much lower than other groups when the treatment time was over 30 s. Referring to the intensity and stability of the plasma (Figure 1E), we chose the He+0.5% O_2_ plasma for use in our following experiments. (Figure 2C) shows apoptosis in the cells detected by flow cytometry 24 h after He+0.5% O_2_ treatment.

### Changes in mitochondrial membrane potential (MMP), lysosomal leakage and activation of the caspase cascades after plasma treatment

**Figure 3:**
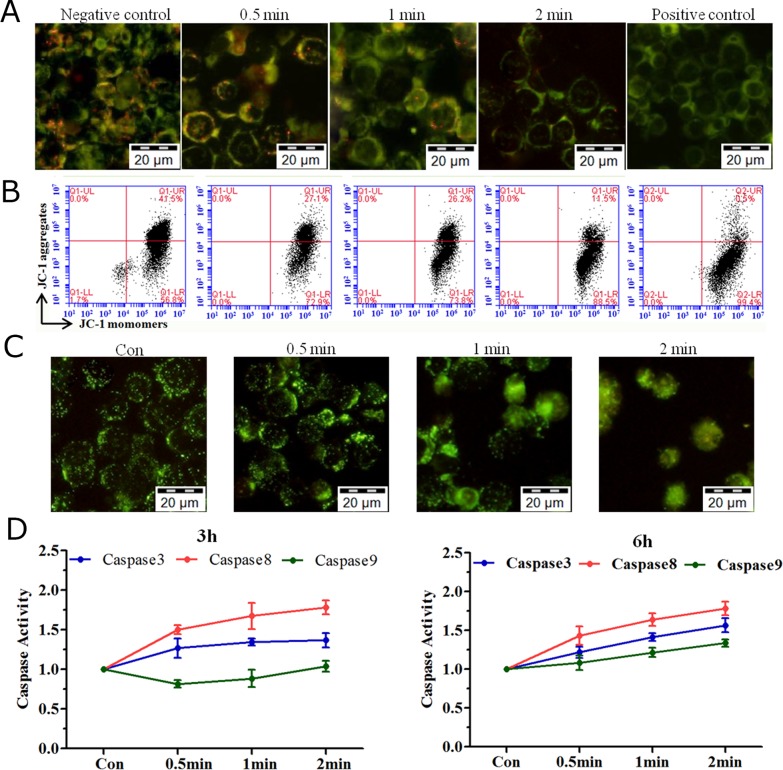
MMP, lysosomal leakage and caspase activation induced by plasma treatment **(A, B)** LP-1 cells stained with a reporter dye, JC-1, for mitochondrial condition are detected by (A) fluorescence microscopy and (B) flow cytometry. **(C)** Analysis of lysosomal leakage in LP-1 cells indicated by fluorescence staining with Lucifer yellow after plasma treatment for 0.5, 1 and 2 min. **(D)** The activity of caspase3/8/9 was measured with a Caspase Colorimetric Assay Kit 3 h and 6 h after plasma treatment.

Changes in MMP (JC-1 staining) are known indicators of early apoptosis [11]. By fluorescence microscopy, we showed that the red fluorescence (JC-1 aggregates) declined after He+O_2_ plasma treatment (Figure 3A), which was further confirmed by flow cytometry (Figure 3B). Lysosomal leakage assay (stained by lucifer yellow dye) showed that plasma treatment resulted in the release of the dye from lysosomes into the cytoplasm, generating diffuse green staining instead of the punctate pattern seen in the control (Figure 3C). Since the activation of caspase cascades plays a central role in apoptosis, we investigated enzyme activity of caspase3/8/9 at 3 h and 6 h after He+O_2_ plasma treatment. The results showed that the enzyme activity of caspase3/8 was increased by plasma treatment, while that of caspase9 increased slightly only at 6 h after treatment (Figure 3D). Western blotting results showed that caspase3/8/9 (32 kD, 31 kD and 18 kD, respectively) were all increased after plasma treatment (Supplementary Figure 1). Cleaved caspase8 (43 kD and 41 kD) exhibited a significant increase, while that of caspase8 (55 kD) remained the same after plasma treatment. The level of caspase9 (47 kD) was slightly increased after plasma treatment. These results indicated that caspase3 and caspase8 were the main factors activating the apoptotic process.

### Analysis of apoptosis-related protein array after He+O_2_ plasma treatment

**Figure 4:**
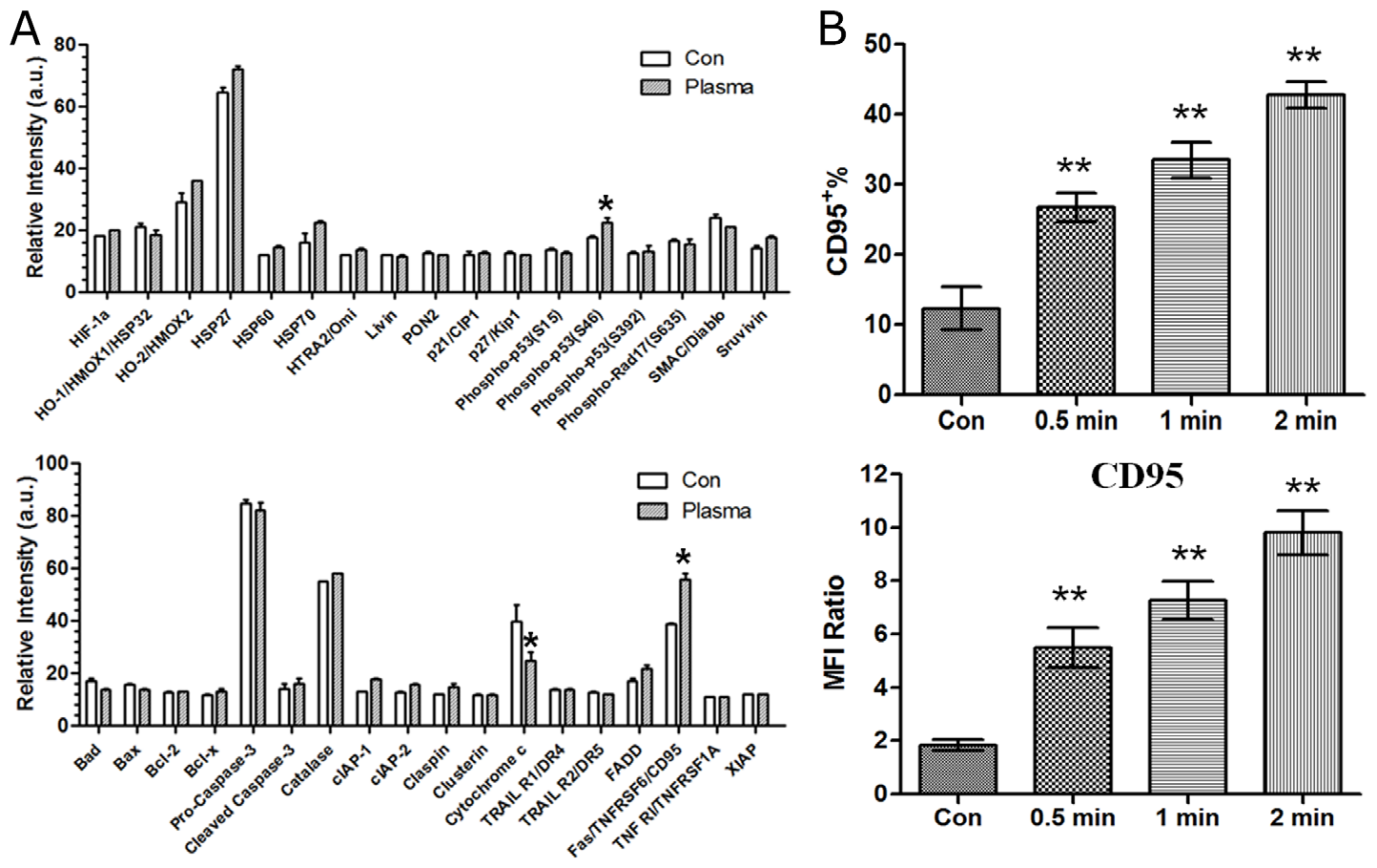
Analysis of apoptosis-related protein array and CD95 expression after plasma treatment **(A)** Cell apoptotic apoptosis-related protein array detection was performed 24 h after He+O_2_ plasma treatment for 1 min. **(B)** CD95 expression was measured by flow cytometry 24 h after He+O_2_ plasma treatment for 0.5 min, 1 min and 2 min. IgG was used as the isotype control of CD95. Percentage of CD95 expression and MFI ratio were expressed in M±SD. ^*^ indicates p<0.05, ^**^ indicates p<0.01

To elucidate the molecular mechanism of plasma-induced cell apoptosis, a human apoptosis protein array containing 35 apoptosis-related proteins was used 24 h after He+O_2_ treatment for 60 s. As shown in (Figure 4A), the level of cytochrome C was decreased, while that of CD95 and phospho-p53 was significantly increased by plasma treatment. As CD95 is a key cell death receptor (DR), we confirmed CD95 expression by flow cytometry after plasma treatment. The results showed that CD95 was up-regulated in a time-dependent manner by He+O_2_ treatment (Figure 4B). These results indicated that CD95 might be involved in plasma-induced cell apoptosis.

### Down-regulation of CD95 reduced plasma-induced cell apoptosis

**Figure 5:**
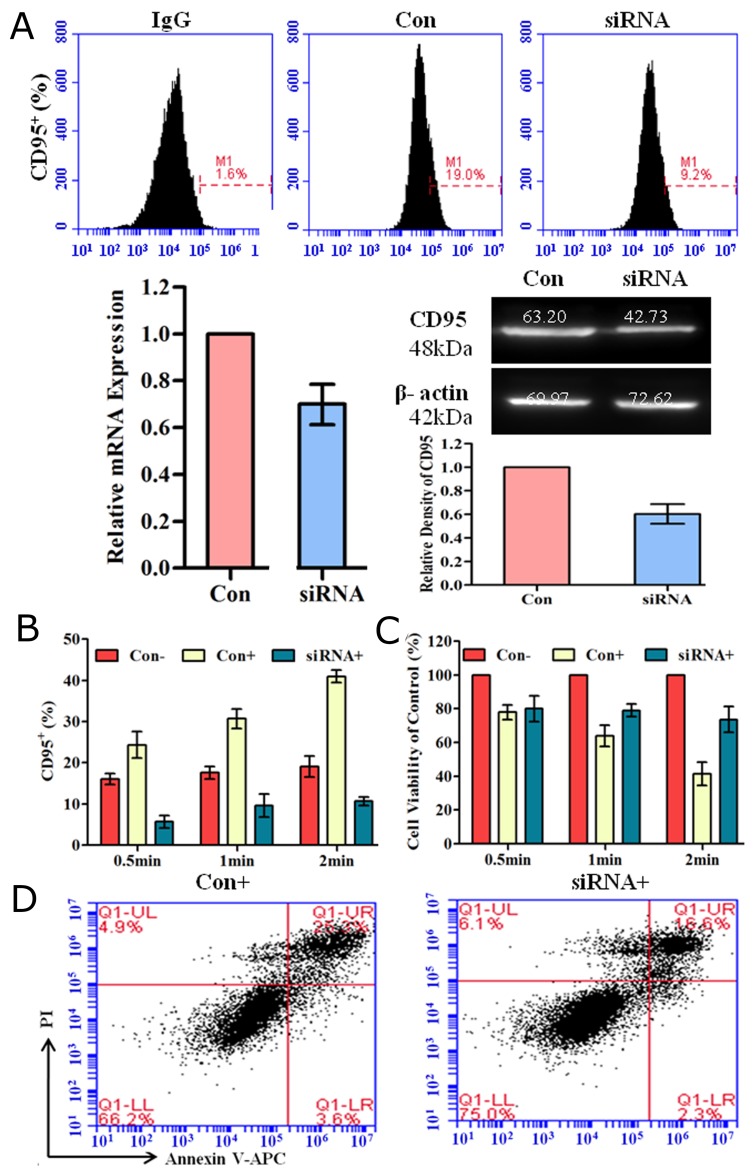
Down-regulation of CD95 expression by siRNA reduced plasma-induced cell apoptosis **(A)** CD95 expression detected by flow cytometry, real-time PCR and western blotting after siRNA-mediated knockdown for 48 h. IgG is the isotype control. Con indicates transfection of control scramble siRNA compared to CD95-targeting siRNA. **(B, C)** CD95 expression (B) and corresponding cell viability (C) 24 h after plasma treatment for 0.5 min, 1 min and 2 min. Con- and Con+ groups were transfected with control scramble siRNA and treated without or with He+O_2_ plasma. The siRNA^+^ group represents cells treated with He+O_2_ plasma after knockdown of CD95 by siRNA. **(D)** Cell apoptosis was detected 24 h after He+O_2_ plasma treatment for 1 min following transfection. ^*^ indicates p<0.05.

We used CD95-targeting short interfering RNAs (siRNA) to down-regulate CD95 expression and assessed the effect of plasma on cell apoptosis. 48 h after siRNA transfection, we detected CD95 expression by flow cytometry, real-time PCR and western blotting. As shown in (Figure 5A), the percentage of CD95^+^ cells was decreased after siRNA transfection. CD95 expression at mRNA and protein levels was also decreased compared to that of the control. The percentage of CD95^+^ cells (Figure 5B) and corresponding cell viability (Figure 5C) after CD95 knockdown and plasma treatment was investigated. CD95 expression and cell viability was negatively correlated. Down-regulating CD95 could reduce cell viability reduction caused by plasma treatment. By Annexin-V/PI staining, we further confirmed that compared to the control, down-regulation of CD95 could reduce cell apoptosis induced by He+O_2_ plasma treatment for 1 min (Figure 5D). These results indicated that CD95 is involved in He+O_2_ plasma-induced cell apoptosis.

### ROS accumulation is essential for CD95-mediated cell apoptosis by plasma treatment

**Figure 6:**
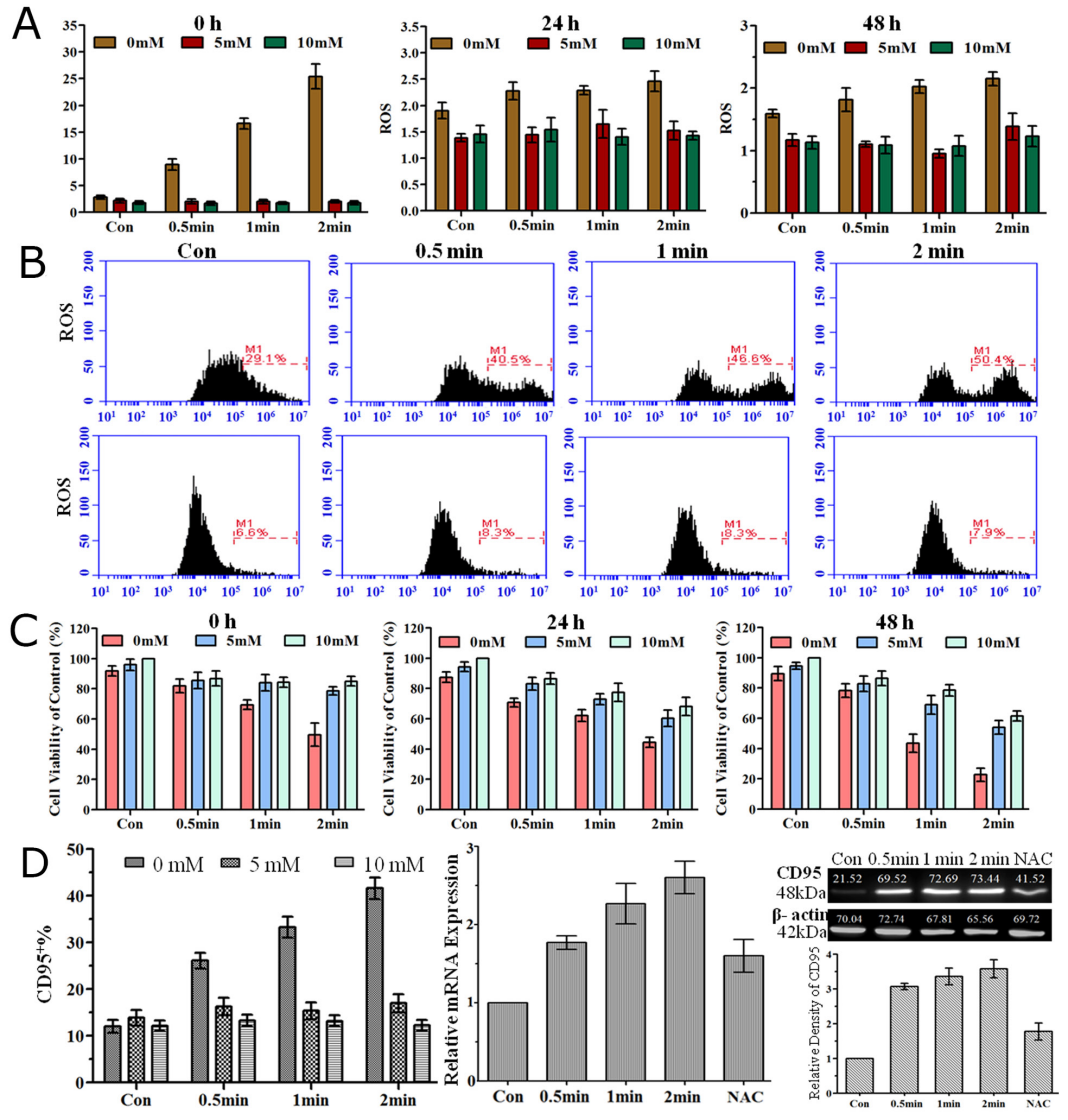
Extracellular and intracellular ROS, cell viability and CD95 expression were measured after He+O_2_ plasma treatment with ROS scavenger (NAC) **(A)** Extracellular ROS level detected with a microplate reader at 0 h, 24 h and 48 h after plasma treatment for 0.5, 1 and 2 min with or without NAC. **(B)** Intracellular ROS level detected with a flow cytometer 24 h after plasma treatment without (above) or with (below) NAC. **(C)** Cell viability measured by CellTiter-Glo assay at 0 h, 24 h and 48 h after plasma treatment for 0.5, 1 and 2 min with or without NAC. **(D)** CD95 expression was detected by flow cytometry (left) and real-time PCR (middle) 24 h after He+O_2_ treatment and by western blot (right) 48 h after plasma treatment with or without NAC.

As ROS produced by plasma are considered to play an important role in biological processes, we investigated extracellular and intracellular levels of ROS after He+O_2_ plasma treatment. Extracellular ROS level was significantly increased immediately after plasma treatment, while at 24 h and 48 h after treatment, it was only slightly higher than the level before treatment (Figure 6A). NAC, which is a general ROS scavenger, could efficiently eliminate extracellular ROS accumulation at a final concentration of 5 mM or 10 mM. Meanwhile, NAC alone (5 mM or 10 mM) had no cell toxicity on LP-1 cells as no reduction of cell viability was detected after adding NAC for 24 h and 48 h. Intracellular ROS was detected by flow cytometry, and the results showed that there was no difference immediately after plasma treatment, or in a short time (3 h and 6 h) after plasma treatment (data not shown), but the level was significantly increased 24 h after plasma treatment (Figure 6B). NAC not only eliminated extracellular ROS but also blocked the intracellular ROS accumulation despite the various durations of plasma treatment. Cell viability assay revealed that preventing ROS accumulation by NAC could reduce plasma-induced cell apoptosis measured at 0 h, 24 h and 48 h (Figure 6C). Furthermore, elimination of extracellular and intracellular ROS accumulation by NAC prevented the increase in the expression of CD95 by plasma treatment measured by flow cytometry, real-time PCR and western blotting (Figure 6D).

### p53 mediated CD95 up-regulation in plasma-induced apoptosis

**Figure 7:**
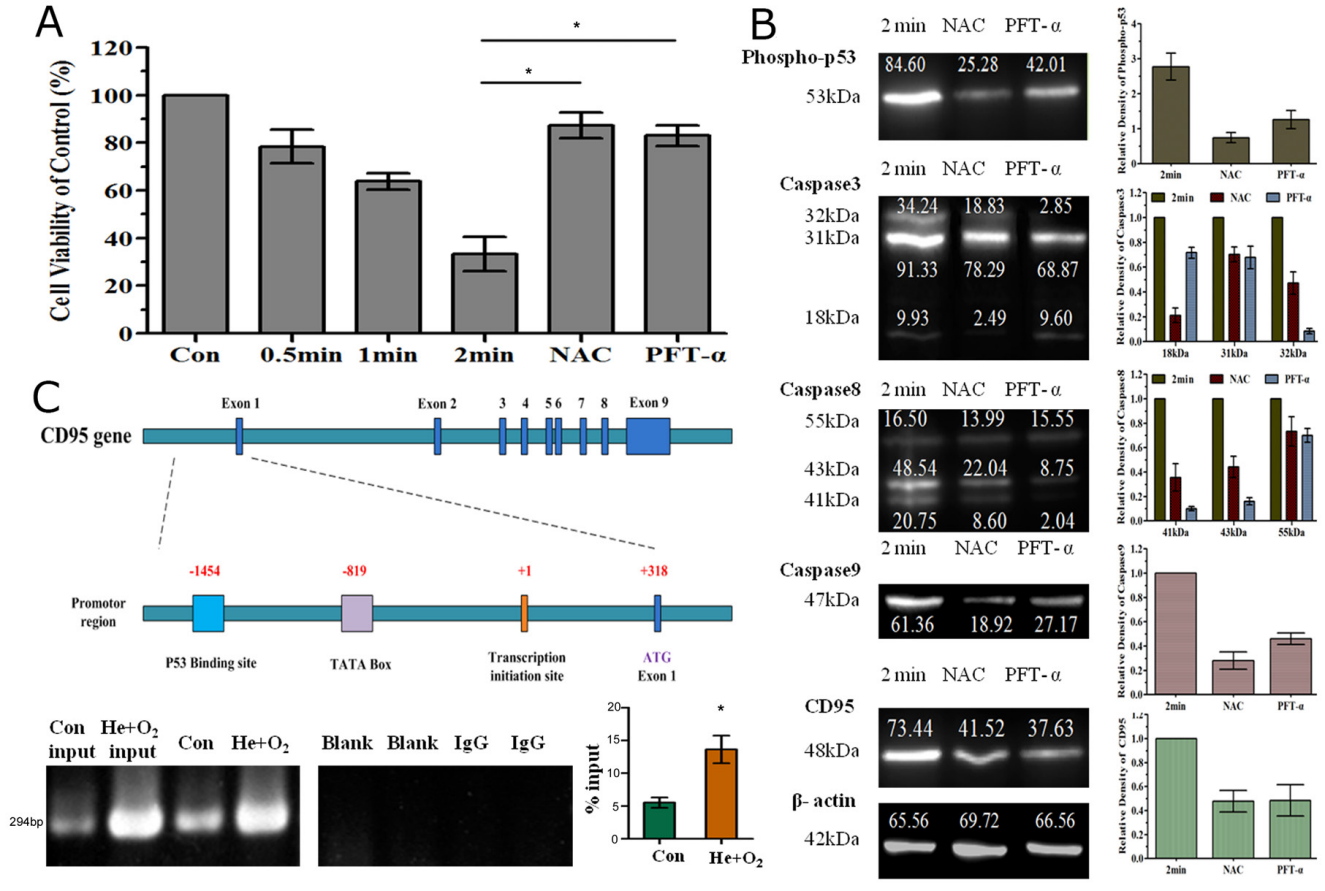
Involvement of p53 in CD95-mediated cell apoptosis by He+O_2_ plasma treatment **(A)** Analysis of cell viability after plasma treatment for 0.5, 1 and 2 min with or without NAC or PFT-α (p53 inhibitor). **(B)** Western blot analysis of phospho-p53, caspase3/8/9 and CD95 expression after plasma treatment. NAC and PFT-α indicates that the ROS scavenger and p53 inhibitor were added respectively before plasma treatment for 2 min. **(C)** Interaction of p53 and CD95 promoter region in response to plasma treatment. The top panel shows the illustration of the CD95 promoter region and p53 binding site. The bottom panel shows the results of ChIP assay for p53 and CD95 promoter detected by RT-PCR and real-time PCR. ^*^ indicates p<0.05.

Using the apoptosis-related protein array we also detected an increase in phospho-p53 level after He+O_2_ plasma treatment. In addition, several studies had reported that p53 was activated in response to plasma treatment and promoted cancer cell apoptosis [36–39]. We wondered whether p53 could regulate CD95 expression thus activating the downstream caspase pathway. PFT-α, a p53 inhibitor, was used in this study and could reduce cell apoptosis by plasma treatment (Figure 7A). Western blotting showed that NAC and PFT-α could both decrease the expression of phospho-p53. Meanwhile, inhibition of p53 prevented the up-regulation of CD95 expression and the activation of caspase 3/8/9 detected by western blotting (Figure 7B). Since p53 is a transcription factor, using ChIP assay, we investigated whether p53 could bind to the promoter region of CD95 to regulate CD95 expression. Indeed, our ChIP results demonstrated that phospho-p53 could bind to the promoter region of CD95 mRNA and regulate mRNA expression, as a clear band was detected by electrophoresis in the sample treated with He+O_2_ plasma (Figure 7C). He+O_2_ plasma increased the binding of p53 to the CD95 promoter region (−1454 upstream of the CD95 transcription initiation site) compared to the control and activated CD95 mRNA expression (Figure 7C). The details of sequence analysis are list in Supplementary Files (sequences analysis.doc).

### Differential expression of CD95 and sensitivity of normal and myeloma cells to plasma treatment in cell lines and clinical samples

**Figure 8:**
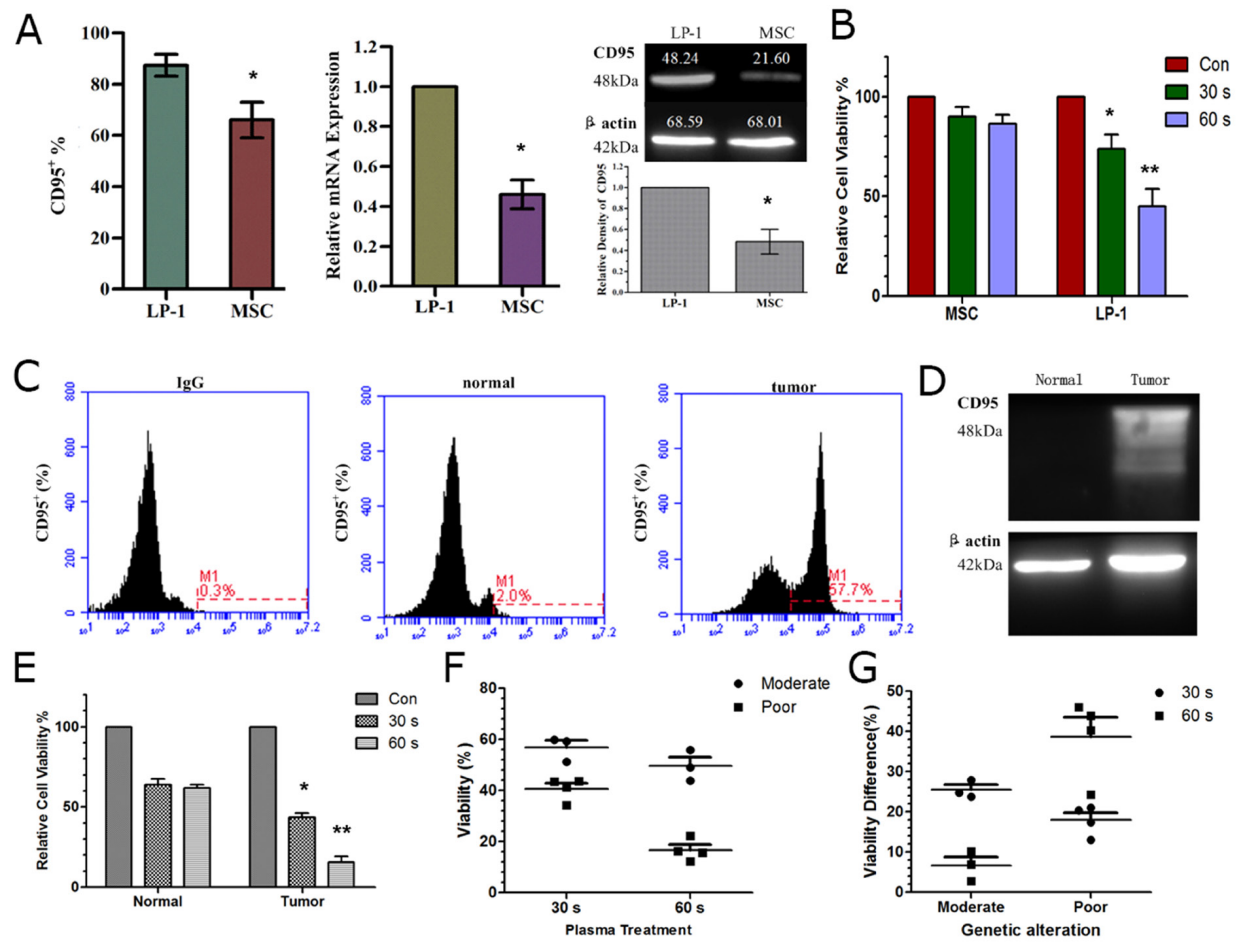
Differential CD95 expression and sensitivity to plasma of cell lines and patient samples **(A)** CD95 expression in MM tumor cells and normal cells (MSC) detected by flow cytometry, real-time PCR and western blotting. **(B)** Sensitivity of tumor and normal cells to plasma treatment assessed by cell viability assay. **(C, D)** CD95 expression detected by flow cytometry (C) and western blotting (D) in a representative patient sample. **(E)** Sensitivity of tumor and normal cells derived from patients assessed by cell viability assay 24 h after plasma treatment. **(F)** Sensitivity of MM tumor cells derived from patients with moderate and poor prognosis (analyzed by FISH) in response to plasma treatment, **(G)** Difference in viability of tumor and normal cells after plasma treatment in samples from patients with moderate and poor prognoses. ^*^ indicates p<0.05, ^**^ indicates p<0.01.

Since we demonstrated that CD95 is an important target in plasma-induced cell apoptosis, we wondered whether normal and tumor cells express different levels of CD95 and if the cells have differential sensitivity to plasma treatment. As shown in (Figure 8A), LP-1 myeloma cells have a higher expression of CD95 compared to normal cells (marrow stromal cells, MSC) detected by flow cytometry, real-time PCR and western blotting. Meanwhile, viability assay revealed that MM tumor cells were more sensitive to plasma treatment that MSC normal cells (Figure 8B). Similar to the data from MM cell lines, the tumor cells derived from MM patients displayed higher CD95 expression compared to normal control detected by flow cytometry (Figure 8C) and western blotting (Figure 8D). In addition, the tumor cells showed a significant reduction in cell viability after plasma treatment compared to the normal control (Figure 8E). By fluorescent in situ hybridization (FISH), we detected several common genetic alterations in MM that could predict the prognosis of MM patients. Patients with RB1 deletion and 13q14 deletion were considered to have moderate prognosis, while patients with 1q21 amplification and 14q32 translocation were considered to have poor prognosis [35] (Supplementary Table 2). Interestingly, tumor cells derived from patients with poor prognosis were more sensitive to plasma treatment than those from patients with moderate prognosis (Figure 8F). Furthermore, we analyzed the viability differences of tumor cells and normal cells after plasma treatment and found that for the samples from patients with moderate prognosis, shorter plasma treatment (30 s) showed better selectivity, while that with poor prognosis, longer plasma treatment (60 s) showed better selectivity (Figure 8G). These data may provide useful treatment parameters for clinical therapy.

## DISCUSSION

Plasmas are a mixture of charged particles, neutral gas molecules, UV radiation, and reactive species, among which, the reactive species were considered to be the most important factors for biological effects [40]. The temperature of plasma in our study is around 30°C, which has little heat effect on cells as cells were further cultured at 37°C in the incubator. This plasma jet generation system was used in our previous studies and characters of the plasma generation and electronic parameters were well illustrated in our previous works, including the distribution of various species by emission spectrum and mass spectrometer [28]. We also demonstrated that plasma jet could generate aqueous reactive species including OH, H_2_O_2_, O_2_^−^ and nitrite (HNO_2_/NO_2_^−^), among which the long-lived species (H_2_O_2_) could reach to μM concentration level [41, 42]. In this study, we found for the first time that CD95/FAS is a pivotal target that induces cancer cell apoptosis in response to plasma treatment. The activation of CD95 was positively correlated with the accumulation of extracellular and intracellular ROS. Interestingly, there was a time lag between the increase in extracellular ROS and the accumulation of intracellular ROS, as the former was significantly increased immediately after plasma treatment, but the latter could only be detected 24 h after plasma treatment. Only some small molecules such as H_2_O_2_ can catalytically permeate cells with the aid of the membrane transporters [43]. However, other ROS, such as OH radicals, cannot pass through the cell membrane owing to their high reactivity. Therefore, most of the intracellular ROS are generated inside the cells through signal transduction from the extracellular ROS. This can explain the time lag for the detection of intracellular ROS 24 h after plasma treatment. However, details regarding signaling transduction by ROS are mostly unknown because of the high reactivity and extreme short life of ROS.

We used He+O_2_ plasma in our study, as the addition of O_2_ can increase the production of ROS by plasma and cause more cell death. From the emission spectra, we confirmed that the He+O_2_ plasma generated more ROS such as OH, O777 and O845 compared to the He plasma. However, higher percentage of O_2_ in the He mixture can decrease plasma intensity or even inhibit plasma generation. Considering the plasma intensity and stability (monitored using electric current) along with its effects on cell viability, we chose the plasma generated from He+0.5% O_2_ for our subsequent experiments. Several studies also reported that He+O_2_ plasma could substantially reduce cancer cell viability [44, 45].

ROS production by other approaches (such as chemo-drugs) can also result in cell apoptosis through up-regulation of CD95, which has been reported in the literature [16, 46]. Here, we demonstrated that ROS production by plasma treatment, could lead to cell apoptosis via the activation of CD95 and downstream caspase cascade. By down-regulation of CD95, we confirmed that CD95 is a major executor in plasma-induced cell apoptosis. However, whether the extracellular ROS can directly affect CD95 to activate the caspase cascade remains unknown. By far, computer modeling could be a way to understand the interaction of small molecules (especially with high reactivity) with biological molecules [47]. In our study, we could only demonstrate with an ROS scavenger that ROS accumulation is necessary for CD95 activation by plasma. Interestingly, we also detected an increase in phospho-p53 in the protein array. The tumor suppressor protein p53 is a redox-active transcription factor that regulates various downstream targets and can induce cell apoptosis [48]. As a secondary messenger, ROS can activate p53 expression, thereby regulating downstream targets of p53 [49, 50]. Zalcenstein et al. reported that in H_2_O_2_-treated human cells, one-third of the highly responsive genes were targets of p53 [51]. Therefore, we investigated whether CD95 up-regulation in response to plasma treatment was mediated by p53. Using anti-phospho-p53 antibodies, we isolated the DNA fragments bound to p53. With well-designed PCR primers against the CD95 promoter region, we observed a significant increase in interaction between p53 and CD95 promoter after He+O_2_ plasma treatment. Furthermore, p53 inhibitor could reduce the up-regulation of CD95 induced by plasma treatment. Hence, for the first time, we reported the details of ROS/p53/CD95/caspase activation with a He+O_2_ plasma treatment system. By ChIP assay, Ruiz et al. reported the presence of p53-mediated up-regulation of CD95 gene expression upon genotoxic treatment in human breast tumor cells, which was consistent with our results showing that p53 could interact with CD95 promoter, thereby regulating its mRNA expression [52].

Unlike radiation, which can penetrate deep inside tissues, CAP has limited penetration. For topological treatment such as in dermatology and wound healing, direct plasma treatment could be applied. For other internal diseases, injection of plasma-activated medium (PAM) could be used. Many studies have reported the induction of cell death by PAM in various tumor cells such as glioblastoma cells, lung adenocarcinoma cells and ovarian cancer cells [53–56]. Understanding the mechanism of plasma-induced myeloma cell death will provide a better strategy for future clinical trials investigating the injection PAM into the blood or bone marrow. However, before gas plasma could be applied in clinical cancer treatment, the safety should also be concerned. It is revealed that plasma treatment could selectively induce tumor cells apoptosis but had little cytotoxicity on normal cells [53, 57]. Our previous study demonstrated that oral lavage of PAW treatment on immuno-deficient nude mice showed no significant safety problems without lethal effect and other acute toxicity [58]. Besides, some gas plasma devices have been applied in clinical trials, especially in wound healing [59, 60]. Some products in plasma medicine have been certified by the U.S. Food and Drug Administration (FDA) [61], although they all limited in skin treatment. In this study, we detected that the myeloma cells were more sensitive to plasma treatment compared to the normal stromal cells (MSC). Since we demonstrated that CD95 is critical in plasma-induced apoptosis, we analyzed the differential expression of CD95 in cancer and normal cells. We found that CD95 expression was higher in tumor cells than in normal cells in both MM cell lines and clinical samples. Interestingly, we predicted prognosis by FISH to detect genetic alterations and found that cells from MM patients with poor prognosis were more sensitive to plasma treatment. This may partly be because patients with poor prognosis have a higher percentage of MM tumor cells in the bone marrow. These results further confirmed that CD95 could be a potential target for myeloma treatment with gas plasma.

In conclusion, we demonstrated that He+O_2_ plasma could efficiently induce cell apoptosis through the activation of CD95 and downstream caspase cascades. Extracellular and intracellular ROS accumulation was essential for CD95-mediated cell apoptosis in response to plasma treatment. Furthermore, p53 was shown to be a key transcription factor in activating CD95 and caspase cascades. Supplementary Figure 2 illustrates the schematic representation of He+O_2_ plasma-induced cell apoptosis mediated by ROS/p53/CD95/Caspase signaling. Meanwhile, we demonstrated that CD95 expression was higher in tumor cells than in normal cells in both MM cell lines and MM clinical samples, which suggests that CD95 could be a favorable target for plasma treatment as it could selectively inactivate myeloma tumor cells.

## MATERIALS AND METHODS

### Plasma generation system

The cold atmospheric plasma used in this study was generated using a plasma jet described in our previous study [62]. And the schematic diagram of the device structure is shown in Figure 1A. A 1 mm stainless steel needle as a powered electrode enclosed in a length of 6 cm and inner and outer diameters of 4 mm and 6mm quartz tube respectively, which is 15 mm above the top of quartz tube. A length of 10mm copper sheet was wrapped around the quartz tube at a distance of 10 mm from the nozzle end serves as the ground electrode. The plasma generation system included a gas flow controller, high-voltage power supply, oscilloscope, and plasma jet. A gas flow of 2 SLM was used for He at 10 kHz/8 kV. Additional O_2_ was added in the gas flow to generate He+O_2_ plasma for more ROS production.

### Cell culture conditions

The LP-1 multiple myeloma cells and MSC cells were used as control cells as described in our previous study [62]. These suspension cells were grown in Roswell Park Memorial Institute (RPMI) 1640 medium supplemented with 10% fetal calf serum, 100 U/mL penicillin, and 50 μg/mL streptomycin (Corning, Ithaca, NY, USA). The cells were cultured at 37°C in an incubator (Thermo Scientific, Waltham, MA, USA) containing 5% CO_2_. The medium was refreshed 24 h before performing experiments, then 6 × 10^4^ cells/ well in 100 μL medium were seeded on 96-well plate and 2 × 10^5^ cells/ well in 300 μL medium were seeded on 24-well plate when performing the plasma treatment experiments.

### Optical emission spectroscopy

Emission spectra of the plasma were measured using a UV/Visible spectrometer (Maya pro 2000, Ocean Optics, China) within a wavelength range of 200-800 nm. The emission spectra of He plasma with different percentage of O_2_ were analyzed in the vertical plane of the plasma jet. Because the intensity of light is highest at the point where the plasma touches air, the optical probe was mounted at the nozzle of the plasma jet generator, which guarantees a clear spectrum of particles in the plasma plume.

### Cell viability assay

A Cell-Titer-Glo^®^ luminescent cell viability assay kit (Promega, Madison, WI, USA) which based on the production of ATP in viable cells was used to measure cell viability. 100 μL of samples and 100 μL of Cell-Titer-Glo^®^ reagent were added to the opaque-walled multi-well plate, then the plate was incubated at room temperature for 10 min after mixing for 2 min on an orbital shaker. The luminescence was determined using the microplate reader ((Thermo Scientific Varioskan Flash, Waltham, MA, USA) with the protocol of “luminometric” measurement.

### Cell apoptosis assessment

We used Annexin V and PI (BioLegend, San Diego, CA, USA) double staining for the detection of cell apoptosis by flow cytometry. After treatment with plasma for 24 h, cells were washed twice with cell staining buffer and resuspended in 50 μL Annexin V binding buffer. Next, 2 μL Annexin V-APC and 2.5 μL PI were added and incubated for 15 min at room temperature in the dark. Then, 400 μL Annexin V binding buffer was added, followed by flow cytometry (Accuri C6; BD, USA).

### Detection of MMP

Mitochondria staining kit (Sigma-Aldrich, Saint Louis, MO, USA), which contains the cationic lipophilic dye JC-1, was used to detect changes in the MMP in LP-1 cells after plasma treatment following the manufacturer's instruction. After incubation for 24 h, 1 mL LP-1 cell suspension (1×10^6^/mL) was collected from each treatment and mixed with 1 mL prepared staining solution with JC-1 at a final concentration of 2.5 μg/mL. The mixture was incubated for 20 min at 37°C in an incubator containing 5% CO_2_. After washing with ice-cold 1× JC-1 staining buffer, the cells were resuspended in 0.5 mL staining buffer. The stained cells were observed by fluorescence microscopy (BX53; Olympus, Japan), following the step of concentrating the suspensions onto microslides by centrifugation for 15 min at 750 rpm with a cytocentrifuge (Cytopro; Wescor, Logan, UT, USA), as well as analyzed directly by flow cytometry (BD). Valinomycin-treated cells were used as positive control, because valinomycin can dissipate the MMP in LP-1 cells.

### Stability of lysosomal membrane

Lucifer yellow CH (Sigma-Aldrich, 2 mg/ml stock solution in PBS), which indicates the stability and leakage of lysosomes, wasadded to LP-cells at a final concentration of 100 μg/ml and incubated overnight. Then, the cells were washed three times with PBS and seeded on a 24-well plate. Next, the stained LP-cells were treated with plasma for 0 min (control group), 0.5 min, 1 min and 2 min. After incubation for 24 h, cells were washed twice and concentrated onto a glass slide using a cytocentrifuge (Cytopro; Wescor, Logan, UT, USA) for observation of the cell status via fluorescence microscopy (BX53; Olympus, Japan).

### Human apoptosis array kit

Human Apoptosis Array Kit (R&D Systems, Minneapolis, MN, USA), which can detect 35 different proteins like Bad, Bax, BCL-2, Fas and HSP27 etc., was purchased for analyzing the expression profiles of apoptosis-related proteins induced by plasma treatment for 1 min. After 24 h incubation, LP-1 cells treated with or without plasma were rinsed with PBS and solubilized in lysis buffer 17 for 30 min at 4°C, followed by centrifuging at 14,000 rpm for 5 min, then the supernate was transferred and protein concentration was quantified. Cell lysates with 300 μg protein was added and incubated overnight at 4°C after blocking membrane with array buffer 1. Following washing each array with 1×wash buffer for 10 min twice, 1.5 mL of detection antibody cocktail was added and incubated 1 h on a rocking platform shaker. Then the array was washed and 2.0 mL of Streptavidin-HRP was pipetted into each well and incubated for 30 min. After washing each array, the protein spots were visualized using Chemi Reagent Mix by Chemi-Doc-it Imaging System (UVP, Upland, CA, USA) and the intensity was determined by ImageJ Version 1.45 software.

### Analysis of CD95 expression by flow cytometry

The expression of CD95 was assayed by immunofluorescence staining using flow cytometry. In brief, LP-1 cells were harvested and washed twice with PBS. Then, the cells were incubated with 5 μL of CD95-FITC (BioLegend, San Diego, CA, USA) in 50 μL PBS for 30 min at room temperature in the dark. After that, the cells were washed with PBS and resuspended in 400 μL PBS for flow cytometric (C6) analysis. Mouse IgG1-FITC (Biolegend, San Diego, CA, USA) was used as an isotype control for CD95 in this experiment. The MFI (mean fluorescence intensity) ratio was determined as follows: MFI ratio = MFI of CD95 expression / MFI of isotype control.

### Transfection with siRNA

Lipofectamine 2000 was used to transfect CD95-targeting siRNA into LP-1 cells to knock down the expression of CD95 following the manufacturer's instructions. Briefly, at the time of transfection, 1×10^5^ cells/well were plated in 24-well plates with culture medium. For each sample, cells were transfected with CD95-targeting siRNA (Santa Cruz, CA, USA) at a final concentration of 125 nM using 6 μL of Lipofectamine 2000 reagent (Invitrogen, Carlsbad, CA, USA). A scramble siRNA was used as control siRNA. After incubation for 48 h, cell viability and cell apoptosis were analyzed, and CD95 expression was determined by real-time PCR, WB and flow cytometry as mentioned. Then, the cells were treated with plasma for further experiments.

### Detection of ROS

In our experiment, CMH2-DCFDA (Invitrogen, Grand Island, NY, USA) was used to detect ROS concentration. Cells (1×10^5^) were plated in 24-well plates with 300 μL complete medium and treated with plasma for 0.5 min, 1 min and 2 min with or without 5 mM/10 mM NAC. After 24 h, 10 μM of CMH2-DCFDA was incubated with the LP-1 cells for 30 min at 37°C. Extracellular ROS was measured with a microplate reader (Thermo Scientific) with excitation/emission at 485/530 nm using the protocol for “Fluorometric” measurement. Then, the cells were washed three times with PBS and collected for analysis of intracellular ROS using a flow cytometer (Accuri C6).

### Enzyme activity of caspase3/8/9

Caspase colorimetric assay kit (Biovision, USA) was used to evaluate caspase activity in LP-1 cells treated with plasma for 0.5 min, 1 min and 2 min. NAC was added to the cells at a final concentration of 10 mM followed by 2 min plasma treatment. After incubation for 6 h and 9 h, cells were resuspended in 50 μL cell lysis buffer and incubated on ice for 10 min. Then, the concentration of protein in the centrifuged supernatant was assayed. Samples with 150 μg protein in 50 μL cell lysis buffer were added to 96-well plates and cultured with 50 μL of 10 mM DTT and 5 μL of 4 mM substrate at 37°C for 1.5 h. The absorbance was read at 400 nm in a microtiter plate reader (Thermo Scientific).

### Western blot

After plasma treatment for 48h, cell protein was extracted using RIPA lysis buffer (Sigma), and the concentration was determined by BCA protein assay kit (Sigma). Protein samples were denatured with 5×protein loading buffer at 95°C for 5min and separated in 12.5% sodium dodecyl sulfate-polyacrylamide gel electrophoresis (SDS-PAGE) (Bio-Rad, Hercules, CA, USA). Subsequently, proteins were transferred to 0.2 μm of polyvinylidene difluoride membranes (Millpore, Billerica, MA, USA) at 70 V for 2.5 h. After blocking with 5% non-fat milk for 2 h at room temperature, the membrane was incubated with primary antibody and then washed with TBST three times. The horseradish peroxidase (HRP)–conjugated secondary antibodies, goat anti-rabbit IgG and anti-mouse IgG (1:2000) (Abgent, San Diego, CA, USA), were added and incubated for 1h on a horizontal orbital shaker. Images were visualized with ECL chemiluminescent substrate (Millipore, Bedford, MA, USA) using a ChemiDoc-It 510 system (UVP, Upland, CA, USA) and protein bands were analyzed by densitometric analysis using ImageJ software. Primary antibody against human included caspase-3 (1:500), caspase-8 (1:500), caspase-9 (1:500), phospho-p53 (1:200) (Cell Signaling Technology, Danvers, MA, USA), CD95 (1:200) (Santa Cruz, CA, USA) and β-actin (1:1000) (Immuno Way, Newark, DE, USA).

### Real-time PCR

To quantify the relative mRNA expression of CD95 (Fas), real-time PCR was performed after plasma treatment (24 h later) or siRNA trasfection (48 h later). Total RNA was extracted from LP-1 cells using EZNA total RNA kit II (Omega Bio-Tec Inc., Doraville, GA, USA). After quantifying with Nano Drop spectrophotometry (BioTek^®^ Instruments Inc., Winooski, VT, USA), 2 μg of total RNA was used for the synthesis of fist strand cDNA using RevertAid first strand cDNA synthesis kit (Thermo Scientific, Waltham, MA, USA), which performed on Applied Biosystems Veriti™ Thermal Cycler (Applied Biosystems, Foster City, CA, USA). All real-time PCR reactions were set up in a 20 μL mixture containing 1/2 volume of 2×QuantiFast SYBR Green PCR MasterMix (Qiagen, Hilden, NRW, Germany), 0.5 μM of primer, 1 μL of cDNA templates and 8 μL of DNAase-RNase Free water. The above reactions were run on Bio-Rad CFX Connect™ Real-time System (Bio-Rad, Foster City, CA, USA) and amplified with a optimized cycling condition: 5 min at 95°C, then 10 s at 95°C and 30 s at 60°C for 38 cycles; the melting curves were obtained by slow heating (0.5°C/s) from 60°C to 95°C. The primers were designed by Shenggong Company (Shanghai, China) and the sequences were showed below: Fas forward, 5′-CCCAGAATACCAAGTGCAG-3′; Fas reverse, 5′-GTGCATTCCTTGATGATTCCA-3′; β-actin forward, 5′-CATGTACGTTGCTATCCAGGC-3′; β-actin reverse, 5′-CTCCTTAATGTCACGCACGAT-3′. The relative expression of CD95 was normalized against a housekeeping gene of β-actin using the ^ΔΔ^Ct method.

### Effect of p53 inhibitor Pifithrin-α on CD95 expression and plasma-induced cell apoptosis

LP-1 cells (1×10^5^/well) were seeded in 24-well plates with 300 μL RPMI 1640 medium and pretreated with 10 μM of PFT-α (Target Mol, Boston, MA, USA), which is an inhibitor of p53, for 1 h at 37°C prior to treatment for 2 min with plasma. After incubation for 48 h, apoptosis was examined by western blotting as previously described.

### ChIP assay

ChIP assay was performed on LP-1 cells with Magna ChIP™ A/G (Millipore; Billerica, MA, USA) following the manufacturer's instructions. Cells (1×10^6^) were treated with or without (control group) He+O_2_ plasma for 1 min; after incubation for 24 h, cells were collected and fixed in 1% formaldehyde for cross-linking. The mixture was incubated at room temperature for 10 min, and 10× Glycine was added to quench the unreacted formaldehyde. After incubation for 5 min, the cells were centrifuged and washed twice with cold 1× PBS. The cell pellet was incubated with 0.5 mL of cell lysis buffer containing 1× protease inhibitor cocktail II for 15 min on ice. Then, the chromatin was isolated by adding 0.5 mL of nuclear lysis buffer containing 1× protease inhibitor cocktail II, followed by sonication to obtain sheared DNA. Chromatin DNA fragments were diluted in 450 μL of dilution buffer containing protease inhibitor cocktail II and precipitated with antibodies against phospho-p53 (1:100) (Cell Signaling Technology) or isotype control IgG including 20 μL of fully resuspended protein A/G magnetic beads at 4°C overnight with rotation. Protein A/G magnetic beads were precipitated with a magnetic separator and washed with low salt, high salt, LiCl and TE Wash Buffer in sequence. Next, the cross-linked protein/DNA complexes were reversed to free DNA by incubating at 62°C for 2 h with shaking in the presence of 100 μL of ChIP elution buffer and 1 μL of proteinase K followed by incubation at 95°C for 10 min. Then, DNA was purified and used as a template for PCR and real-time PCR using the following primer pairs: 5’-GGATAATTAGACGTACGTGGGC-3’ (forward) and 5’-GGACAATTGACAAAATCAGTATC-3’ (reverse) [63]. The PCR products were detected on a 2% agarose gel.

### Preparation and analysis of MM clinical samples

Seven newly diagnosed MM patients were chosen from the Department of Hematology, Xijing Hospital of Xi’an. The basic information on these patients is listed in Supplementary Table 1, including gender, age and blood test results. Bone marrow samples were also obtained and tested using Giemsa staining to measure the percentage of MM tumor cells. MM tumor cells were sorted using MACS (magnetic-activated cell sorting) from the bone marrow samples. Briefly, 20 mL bone marrow (with heparin) was centrifuged and washed with PBS and resuspended in 7 mL of Ficoll (lymphocyte separation medium). The cells were centrifuged at 1800 rpm for 20 min, and the middle layer was isolated for staining with CD138-conjugated magnetic beads. After incubation at 4°C for 15 min, the cells were washed with PBS and sorted with MACS columns. The positively selected cells were considered MM tumor cells, and the rest of the cells mostly were bone marrow stromal cells that were considered as the control group. The sensitivity treatment of these cells to plasma was detected by a cell viability assay, and CD95 expression was measured by flow cytometry and western blotting.

### Genetic alterations in MM patients detected by FISH

To evaluate the prognosis of MM patients, a FISH kit was used to detect gene abnormalities in MM. The kit can detect several common genetic abnormalities in MM, including RB1 deletion, 13q14 deletion, 1q21 amplification and 14q32 translocation (Supplementary Table 2). FISH was performed according to the manufacturer's instructions; the results were analyzed using the ratio of the number of positive cells to the total number of cells (counting for more than 200).

### Statistical analysis

All samples were prepared in triplicate, and experiments were repeated at least three times. The data are presented as the means ± SD. Differences between groups were evaluated using one-way ANOVA and Student's *T* test. P < 0.05 was considered statistically significant.

## SUPPLEMENTARY MATERIALS FIGURES AND TABLES





## Abbreviations

MM: Multiple myeloma
PCs: Plasma cells
BM: bone marrow
ROS: Reactive oxygen species
DR: Death receptors
TNF: Tumor necrosis factor receptor
ER: Rndoplasmic reticulum
CAP: Cold atmospheric plasma
MMP: Mitochondrial membrane potential
PAM: Plasma-activated medium
MSC: Marrow stromal cells
DBD: Dielectric barrier discharger
FDA: Food and drug administration
RPMI: Roswell Park Memorial Institute
siRNA: Short interfering RNAs
MFI: Mean fluorescence intensity
SDS-PAGE: Sodium dodecyl sulfate-polyacrylamide gel electrophoresis
HRP: Horseradish peroxidase
ChIP: Chromatin immunoprecipitation
MACS: Magnetic-activated cell sorting
FISH: Fluorescent in situ hybridization.
