# Supplementary material for: Cold atmospheric plasma as a potential tool for multiple myeloma treatment
Source: Oncotarget. 2018 Apr 6;9(26):18002–17. doi: 10.18632/oncotarget.24649 (PMC5915053; doi:10.18632/oncotarget.24649)
Supplement: Supplementary file 1 [file oncotarget-09-18002-s001.pdf]

Cold atmospheric plasma as a potential tool for multiple myeloma treatment

SUPPLEMENTARY MATERIALS

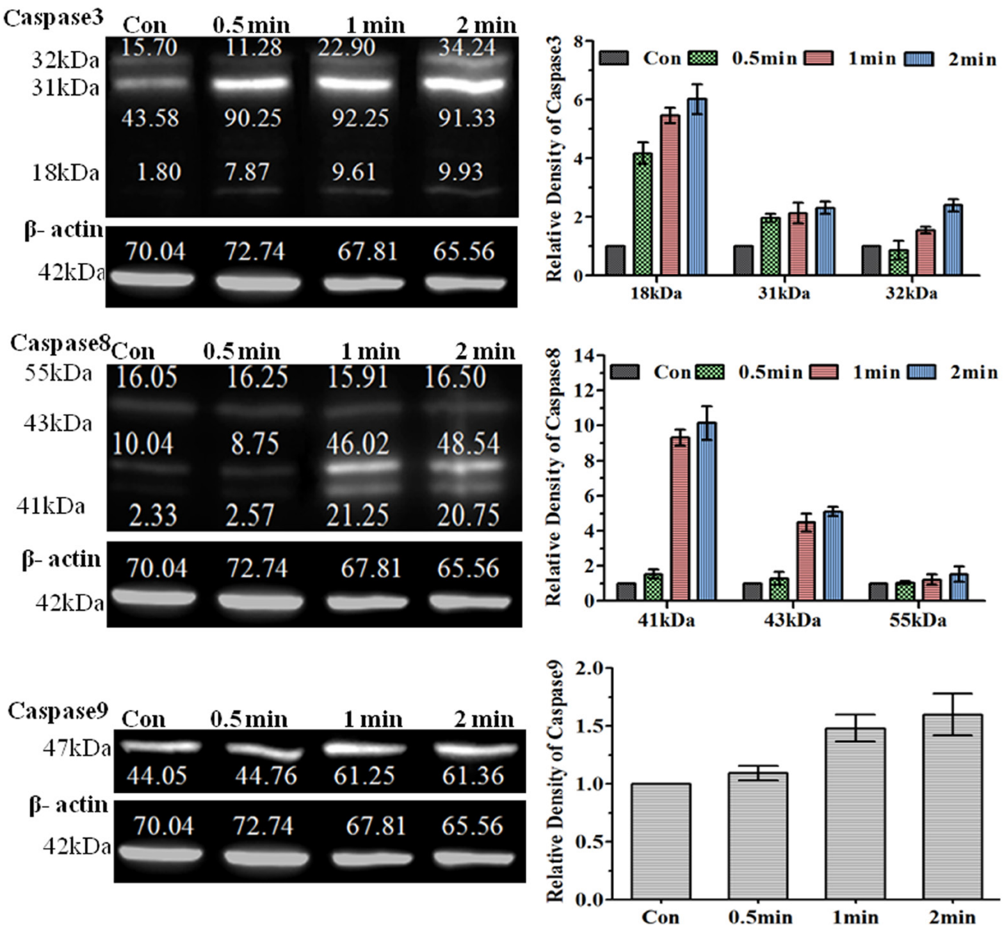

Supplementary Figure 1: Western blot analysis of caspase3/8/9 after He+O_2 plasma treatment for different durations. Con indicates the control group that was treated with gas flow without discharging. NAC indicates that the ROS scavenger NAC was added before plasma treatment for 2 min. Representative results of three independent experiments are shown. Gray value analysis of each band by Image-Pro-Plus software; the expression level was calculated for each caspase band compared to the level of actin as the loading control.

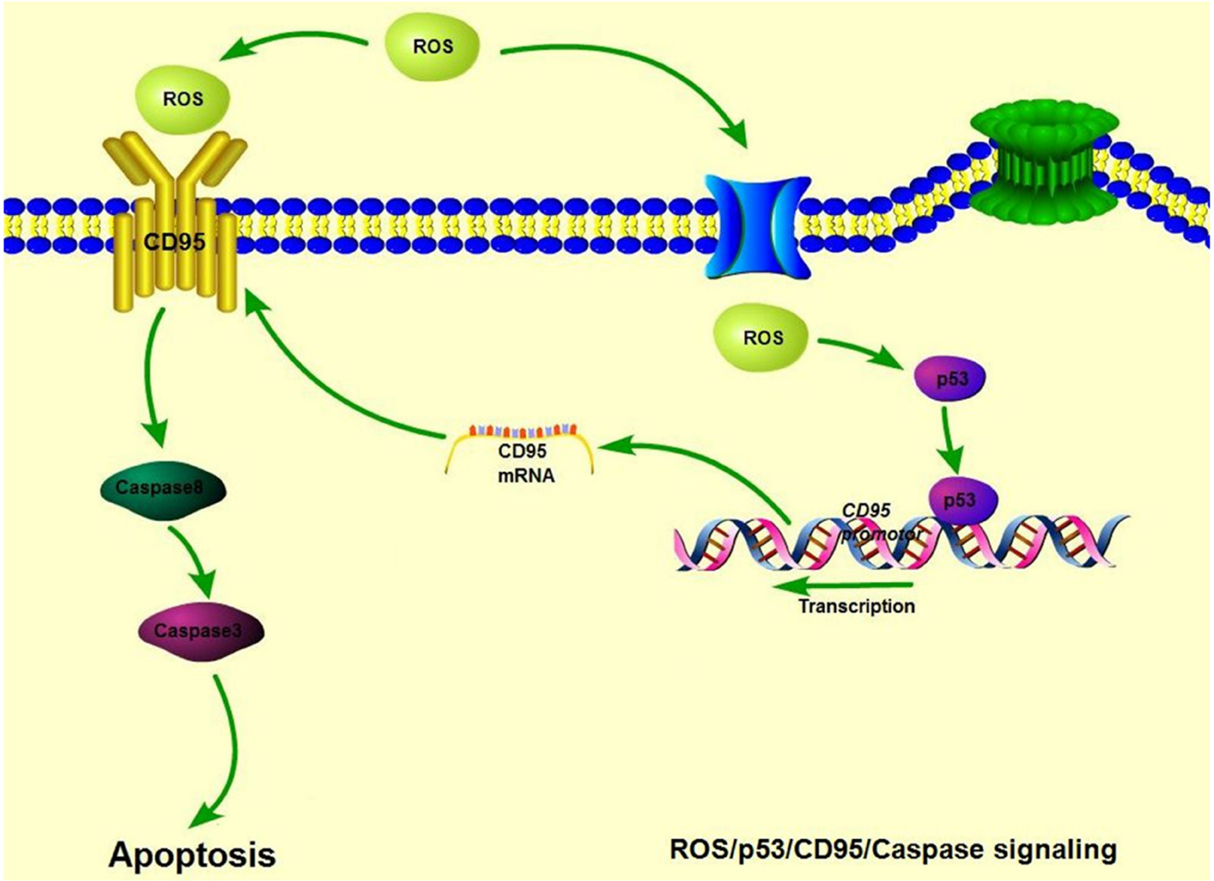

Supplementary Figure 2: Illustration of ROS/p53/CD95/Caspase signaling in response to He+O_2 plasma treatment.

Supplementary Table 1: General information and blood tests of MM patients

Patients	Gender	Age	Percentage of MM cells	WBC (10^9/L)	HB (g/L)	Plt (10^9/L)
Pt1	F	67	20%	8.75	152	228
Pt2	M	40	60%	2.28	76	51
Pt3	M	59	56%	7.13	105	168
Pt4	M	64	30%	6.65	82	128
Pt5	F	66	30%	3.8	99	200
Pt6	M	62	78%	7.66	68	71
Pt7	M	41	60%	6.73	65	43

Supplementary Table 2: Genetic alteration of MM patients detected by FISH analysis

Patients	RB1 Deletion	13q14 Deletion	1q21 Amplification	14q32 Translocation
Pt1	83.5%	79.5%	0	0
Pt2	0	0	66.5%	61.5%
Pt3	61.5%	76%	84.5%	63.5%
Pt4	75.5%	69%	2%	0
Pt5	91.5%	93%	0	56.5%
Pt6	0	0	0	63.5%
Pt7	91.5%	88.5%	94%	79.5%

Sequences Analysis: The promoter sequence of CD95. The numbers in red at the beginning and the end presents the exact site of this sequence in chromosome 10 entire DNA "10q23.31". The sequences in purple indicated PCR primers designed for ChIP analysis. Yellow block indicated the p53 binding site. Turquoise block indicated the TATA Box. Pink block indicated the transcription initiation site. Green block indicated the translation starting code ATG.

See Supplementary File 1
