# Supplementary material for: Cold atmospheric plasma as a potential tool for multiple myeloma treatment
Source: Oncotarget. 2018 Apr 6;9(26):18002–17. doi: 10.18632/oncotarget.24649 (PMC5915053; doi:10.18632/oncotarget.24649)
Supplement: Supplementary file 2 [file oncotarget-09-18002-s002.docx]

[88988958]GGATAATTAGACGTACGTGGGCAGAGGGTAGGGGAAGGGGGTATGGCATAGAAAGAGCAGGACCTTGGGAGCAAGAATATCTAAGTTTAATTCCTGACTCTGCTATTTATTAACTAACCATCTTTGCCAATGTTGCTTAAGCTTTTTTGGCTACATTTTTTTATTTGTAAAGTAAGTTTAATAATCACTCATCTCACTGGGCTATAATGATAAGTATTAAGTAAGGAAGATCCACATATGTGAGTTGCTGGCTTATAATTCACACTCAAGAGATACTGATTTTGTCAATTGTCCTTTCCCCTTTTTTTCTCTCTTCCCTCCTTCCATTCCTTCTTCCCTTACCTCTCCTTTCCTTCCCTCACACCCCTTTTCCTTCCTTCTTTTTACATTTTTTTATTTAAATGAACTTTTCATTTTGGAATAGTTTTAGGATTTCAAAAAATTTGCAGAGATAATACAGAGAATGCCCATATACCATCCTCCTTATCCCACTTCTTTTTGTGTCTATTAGATGCTCAGAGTGTGTGCACAAGGCTGGCACGCCCAGGGTCTTCCTCATGGCACTAACAGTCTACTGAAAGGTGGAACAGAGACAAGCCTATCAACACCTACAAGACTGGTGGTAAGTGCAGTGACAGATGCAAAACACAGGGTGATGGAAAGCCCTCAGGAGGGTAACCTAACCTAGATTTGAGGGCCCAAACAGGCTCCAGAAGAAAATGTCAACTGAGAGGAAGCCTGAAGGATGAACAGTGGGCTAAGCAAAGGGTTATTAATGTGTTATTAATGGGTTGAATCTAATTGGGAAGGGAGAGAGGTTGCAGAGTGAGGTGCAGAGCTTGGTGGACGATGCCAAAGGAATACTGAAACCTTTAGTGTGTCCAGTCTGGAACTGCATCCAAATTCAGGTTCAGTAATGATGTCATTATCCAAACATACCTTCTGTAAAATTCATGCTAAACTACCTAAGAGCTATCTACCGTTCCAAAGCAATAGTGACTTTGAACAGTGTTCACCAGAGCACGAAAGAATTACAAGATTTTTTTTTAAAGAAAATTGGCCAGGAAATAATGAGTAACGAAGGACAGGAAGTAATTGTGAATGTTTAATATAGCTGGGGCTATGCGATTTGGCTTAAGTTGTTAGCTTTGTTTTCCTCTTGAGAAATAAAAACTAAGGGGCCCTCCCTTTTCAGAGCCCTATGGCGCAACATCTGTACTTTTTCATATGGTTAACTGTCCATTCCAGAAACGTCTGTGAGCCTCTCATGTTGCAGCCACAACATGGACAGCCCAGTCAAATGCCCCGCAAGTCTTTCTCTGAGTGACTCCAGCAATTAGCCAAGGCTCCTGTACCCAGGCAGGACCTCTGCGCTCTGAGCTCCATTCTCCTTCAAGACCTCCCCAACTTCCCAGGTTGAACTACAGCAGAAGCCTTTAGAAAGGGCAGGAGGCCGGCTCTCGAGGTCCTCACCTGAAGTGAGCATGCCAGCCACTGCAGGAACGCCCCGGGACAGGAATGCCCATTTGTGCAACGAACCCTGACTCCTTCCTCACCCTGACTTCTCCCCCTCCCTACCCGCGCGCAGGCCAAGTTGCTGAATCAATGGAGCCCTCCCCAACCCGGGCGTTCCCCAGCGAGGCTTCCTTCCCATCCTCCTGACCACCGGGGCTTTTCGTGAGCTCGTCTCTGATCTCGCGCAAGAGTGACACACAGGTGTTCAAAGACGCTTCTGGGGAGTGAGGGAAGCGGTTTACGAGTGACTTGGCTGGAGCCTCAGGGGCGGGCACTGGCACGGAACACACCCTGAGGCCAGCCCTGGCTGCCCAGGCGGAGCTGCCTCTTCTCCCGCGGGTTGGTGGACCCGCTCAGTACGGAGTTGGGGAAGCTCTTTCACTTCGGAGGATTGCTCAACAACCATGC[88990877].

The numbers in red at the beginning and the end presents the exact site of this sequence in chromosome 10 entire DNA "10q23.31".

The sequences in purple indicated PCR primers designed for CHIP analysis.

Yellow block indicated the p53 binding site.

Turquoise block indicated the TATA Box.

Pink block indicated the transcription initiation site.

Green block indicated the translation starting code ATG.
